# Does Molecular and Structural Evolution Shape the Speedy Grass Stomata?

**DOI:** 10.3389/fpls.2020.00333

**Published:** 2020-04-21

**Authors:** Yuanyuan Wang, Zhong-Hua Chen

**Affiliations:** ^1^College of Agriculture and Biotechnology, Zhejiang University, Hangzhou, China; ^2^School of Science, Western Sydney University, Penrith, NSW, Australia; ^3^Hawkesbury Institute for the Environment, Western Sydney University, Penrith, NSW, Australia; ^4^Collaborative Innovation Centre for Grain Industry, College of Agriculture, Yangtze University, Jingzhou, China

**Keywords:** epidermal patterning, guard cell signaling, molecular breeding, stomatal development, stomatal structure

## Abstract

It has been increasingly important for breeding programs to be aimed at crops that are capable of coping with a changing climate, especially with regards to higher frequency and intensity of drought events. Grass stomatal complex has been proposed as an important factor that may enable grasses to adapt to water stress and variable climate conditions. There are many studies focusing on the stomatal morphology and development in the eudicot model plant *Arabidopsis* and monocot model plant *Brachypodium*. However, the comprehensive understanding of the distinction of stomatal structure and development between monocots and eudicots, especially between grasses and eudicots, are still less known at evolutionary and comparative genetic levels. Therefore, we employed the newly released version of the One Thousand Plant Transcriptome (OneKP) database and existing databases of green plant genome assemblies to explore the evolution of gene families that contributed to the formation of the unique structure and development of grass stomata. This review emphasizes the differential stomatal morphology, developmental mechanisms, and guard cell signaling in monocots and eudicots. We provide a summary of useful molecular evidences for the high water use efficiency of grass stomata that may offer new horizons for future success in breeding climate resilient crops.

## Introduction

Global food demand and consumption is at historically high levels and the current level of crops may not be sustainable if their production is not able to keep up with the population growth and adapt to the changing climate (FAO et al., 2019). Therefore, future molecular and conventional crop breeding approaches are suggested to aim at new cultivars that can maximize yield under a capricious climate (Bailey-Serres et al., 2019; Faralli et al., 2019). One of the positive climate adaptation strategies for agriculture is irrigation, which allows crops to buffer against climate variability (Lobell et al., 2009). However, over-irrigation depletes groundwater and diminishes surface water supplies (Zhu et al., 2019). Therefore, water use efficiency (WUE), the ratio of carbon gains to water use, directed by photosynthesis and gas exchange level, becomes one of the most important challenging targets for crop improvement (Leakey et al., 2019).

Drought is one of the most detrimental abiotic stresses, threatening sustainable food production (Lesk et al., 2016). Water deficit in plants is caused by insufficient soil water availability and high vapor pressure deficit, resulting in a change in plant water status and restricting plant development and productivity (Tardieu et al., 2018). As drought tolerance is a complex trait, breeding for drought tolerance by targeting single genes has not resulted in significant success so far (Tester and Langridge, 2010; Tardieu et al., 2018). Therefore, it is vital to combine quantitative trait loci (QTL) identification, gene pyramiding, genome editing, and other molecular breeding technologies to study fundamental phenotypic traits (e.g., deep root, efficient metabolism for desiccation tolerance) in order to improve WUE and drought tolerance of crops.

Stomatal opening facilitates CO_2_ uptake and water loss in plants. However, the increased transpiration rate will induce stomatal closure to stabilize transpiration (Mott and Parkhurst, 1991; Hepworth et al., 2018). This feedback loop is an important short-term physiological mechanism triggered by plants under water stress (Martínez-Vilalta and Garcia-Forner, 2017). Therefore, understanding the mechanisms of stomatal movement is crucial in regulating plant performance under the forthcoming predicted increasing frequency and intensity of droughts across the globe (Barral, 2019). Furthermore, stomatal movement becomes an obvious target for breeding crops with high WUE and drought tolerance (Lawson and Blatt, 2014; McAusland et al., 2016).

Numerous studies have shown that stomatal structure affects plants’ response to the environmental cues (Hetherington and Woodward, 2003; Franks and Farquhar, 2007; Berry et al., 2010). Species of the grass (Poaceae) family have distinctive dumbbell-shaped guard cells (GCs) and specialized subsidiarry cells (SCs), forming an efficient stomatal complex (Franks and Farquhar, 2007; Chen et al., 2017). For example, light can induce faster stomatal opening in grass species such as barley (*Hordeum vulgare*) and sugarcane (*Saccharum officinarum*) than those in eudicots like broad bean (*Vicia faba*) and soybean (*Glycine max*) (Grantz and Assmann, 1991; Kaiser and Kappen, 1997). Moreover, wheat (*Triticum aestivum*) has significantly faster stomatal opening than *Tradescantia virginiana*, *Nephrolepis exaltata*, and *Huperzia prolifera* (Franks and Farquhar, 2007). In the 1970s, it was proposed that the potassium shuttling between guard cells and subsidiary cells may be the key mechanism for rapid stomatal opening in grasses (Raschke and Fellows, 1971). Later, researchers found the effect of blue-light stimulus on rapid opening of grass stomata (Johnsson et al., 1976; Karlsson and Assmann, 1990; Grantz and Assmann, 1991; Assmann and Jegla, 2016), which explained the mechanical advantage and osmotic shuttling of grass stomata. These characteristics allow for a faster grass stomata response than any other stomatal types. Thus, it was proposed that water and resource utilization have been optimized in grass-specific stomatal complexes during evolution (Franks and Farquhar, 2007). In recent years, there have been huge advancements in the genome sequencing and funcational analysis of genes for stomatal regulation, epidermal patterning, and stomatal development (Raschke and Fellows, 1971; Blatt, 2000; Nadeau and Sack, 2002; Hetherington and Woodward, 2003; Shpak et al., 2005; Abdulrahaman et al., 2009; Berry et al., 2010; Facette and Smith, 2012; Pillitteri and Torii, 2012; Drake et al., 2013; Kollist et al., 2014; Raven, 2014; Cai et al., 2017; Rudall et al., 2017; Han and Torii, 2019). However, the molecular evolution of key gene families for stomatal development and the distinction between eudicot and monocot stomatal structure across a comprehensive set of plant species, representing the major lineages of angiosperms, have not been fully investigated.

This review highlights the unique morphological structure and developmental process of grass stomata and summarizes the contribution of *Arabidopsis* homologous genes in a large number of eudicot and monocots, including grasses. We found that some gene families involved in the lineage-specific stomatal file specification or polarization have certain levels of distinction between monocots and eudicots. We hypothesized that relevant gene families [e.g., SPCH, MUTE, FAMA (SMFs), Breaking of Asymmetry in the Stomatal Lineage (BASLs), and PANGLOSS (PANs)] determine stomatal structure and development, which may further influence stomatal movement and potentially regulate WUE in plants (Chen et al., 2017; Li et al., 2017; Lawson and Vialet-Chabrand, 2018). For more comprehensive reviews of stomatal development and function, the readers are directed to the following excellent articles (Qu et al., 2017; Hepworth et al., 2018; Faralli et al., 2019; Lawson and Vialet-Chabrand, 2018; Tardieu et al., 2018; Leakey et al., 2019).

## Classification of Plant Stomatal Morphology

**FIGURE 1:**
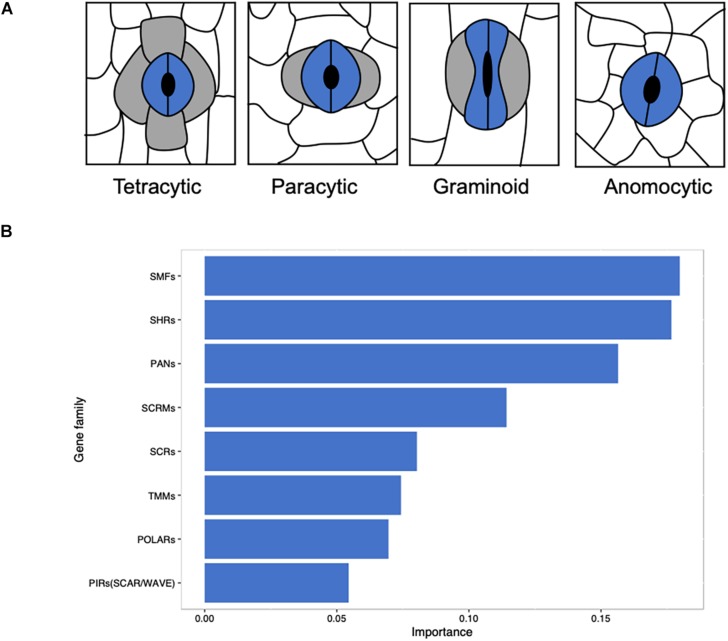
Linking stomatal morphology to the evolution of key gene families in angiosperms. **(A)** Morphological classification of stomata in monocots based on the presence and number of subsidiary cells. Anomocytic without obvious SCs; paracytic with two lateral subsidiary cells; graminoid is a special form of paracytic with two dumbbell-shape guard cells and two lateral subsidiary cells; tetracytic with two lateral subsidiary cells and two polar subsidiary cells. **(B)** A total of 411 eudicots and monocots in OneKP project were use in this study and species with graminoid stomata were separated from other species. Predicted putative sequence files of gene families that are relevant to stomatal development were obtained from OneKP Orthogroups Extractor (http://jlmwiki.plantbio.uga.edu/onekp/v2/), the number of genes of each family in each species was counted. With the distribution of gene families, the contribution of gene families that cause graminoid stomata specifically was computed by Xgboost (R package) with default parameters. Gene families with an importance of more than 0.05 were plotted.

Based on the existence and position of lateral subsidiary cells (LSCs) and shape of GCs, stomata can be divided into seven major morphological classes: anomocytic (no obvious SCs), actinocytic complexes (a circle of radiating SCs), paracytic (LSCs), graminoid (dumbbell-shaped GCs with LSCs), tetracytic (LSCs and polar SCs), diacytic (perpendicular SCs), and cyclocytic (four or more similarly sized SCs) (Abbe et al., 1951; Frynsclaessens and Van Cotthem, 1973; Nunes et al., 2019). Mature monocot stomatal complex is classified into anomocytic, paracytic, and tetracytic (Rudall et al., 2017) and graminoid is a special type of paracytic (Figure 1A). A majority of the studies on stomatal function are conducted on ‘kidney-shaped’ GCs in most eudicots (Nadeau and Sack, 2002; Hamel et al., 2006; Macalister et al., 2007; Lampard et al., 2008; Hara et al., 2009; Pires and Dolan, 2010; Zhang et al., 2015; Houbaert et al., 2018) and some are on the ‘dumbbell-shaped’ stomatal complex in grasses (Cartwright et al., 2009; Raissig et al., 2016, 2017; Chen et al., 2017; Hughes et al., 2017; Hepworth et al., 2018; Caine et al., 2019; Dunn et al., 2019). The stomatal complex of grass species may have facilitated their evolutionary success (Franks and Farquhar, 2007; Cai et al., 2017), resulting in better adaptation under water deficient environments with significant biomass production (Lawson and Vialet-Chabrand, 2018). Dumbbell-shaped GCs are a characteristic structure of *Poaceae* (Stebbins and Shah, 1960), which provides these species with faster responses to environmental changes (Franks and Farquhar, 2007; Cai et al., 2017; Chen et al., 2017) and a closer morphological and physiological connection between GCs and SCs than other monocots and eudicots (Frynsclaessens and Van Cotthem, 1973; Tomlinson, 1974; Rudall et al., 2013; Rudall et al., 2017).

## Stomatal Development Process and Regulatory Models

Stomatal development and epidermal patterning have been extensively studied mainly in *Arabidopsis*, *Brachypodium*, maize, and moss (Nadeau and Sack, 2002; Cartwright et al., 2009; Chater et al., 2017; Raissig et al., 2017; Houbaert et al., 2018). Indeed, monocots and eudicots have huge differences in stomatal development and epidermal patterning. In eudicots, protodermal cell produces meristemoid mother cell (MMC), which asymmetrically divides into stomatal lineage ground cell (SLGC) and meristemoid (M). Meristemoids subsequently differentiate into guard mother cells (GMCs) through asymmetrical division, and division of GMCs produces Young Guard Cells (YGCs). After cell expansion and pore formation, YGCs develop into Mature GCs (MGCs). Moreover, SLGC is another initial point as it can reversely generate another stomatal lineage precursor (M + SLGC) or form a pavement cell (Peterson et al., 2010; Pillitteri and Torii, 2012; Vatén and Bergmann, 2012; Han and Torii, 2016, 2019). Most eudicots, including *Arabidopsis*, have random GC orientation and epidermal patterning (Zhao and Sack, 1999). However, the formation of grasses’ LSCs is still not fully understood due to the complex structure and underlying molecular mechanisms. It was suggested that GMCs induce MGC formation via symmetric divisions and subsidiary mother cell (SMC) via asymmetric divisions in grasses, resulting in divergent stomatal morphology (Raissig et al., 2016, 2017; Hepworth et al., 2018). On the contrary, polar subsidiary cells of tetracytic stomata are strictly mesogene cells because they initiated from stomatal cell files (Rudall et al., 2017; Nunes et al., 2019). It is also worth noting that not all the stomatal developmental patterns are well-ordered and axially polarized among monocots. For example, stomatal orientation is transverse in some *Stemona* and *Lapageria* species, but is random in some *Araceae* and *Dioscorea* species (Abdulrahaman et al., 2009; Rudall et al., 2017).

The extrinsic and intrinsic signals regulate the stomatal development and epidermal patterning in plants and most of the studies employed the model plant *Arabidopsis* (Peterson et al., 2010; Pillitteri and Torii, 2012; Han and Torii, 2019). The heterodimers of basic Helix-Loop-Helix (bHLH) transcription factors specify stomatal precursor cell states (Weintraub et al., 1991). In different developmental stages, two universal components, SCRM/ICE1 and SCRM2, bind with different members of bHLH subgroup Ia SPEECHLESS (SPCH), MUTE, and FAMA, which were recently renamed as SMFs (SPCH, MUTE, FAMA) (Qu et al., 2017). The interactions of ICE1/SCRM2 and SPEECHLESS (SPCH), MUTE, and FAMA promote MMC, GMC and MGC stages respectively (Ohashi-Ito and Bergmann, 2006; Macalister et al., 2007; Pillitteri et al., 2007; Kanaoka et al., 2008). The leucine-rich repeat receptor (LRR) kinase complex that includes receptor-like protein Too Many Mouths (TMM), ERECTA family (ER), and Somatic Embryogenesis Receptor Kinase (SERK) are the primary receptors that transduce extrinsic signals (Shimada et al., 2000; Nadeau and Sack, 2002; Lukowitz et al., 2004; Shpak et al., 2005; Meng et al., 2015). LRR family transduces the developmental signal from EPFs. TMM is a signal modulator that establishes ligand-receptor pairs EPF2-ERECTA and EPF1-ERL1 to specify stomatal developmental initiation and spacing division (Lee et al., 2012). The Mitogen-Activated Protein Kinase (MAPK) cascade, including the MAPKKK/Embryo Defective71 (YODA), MPKK4/5, and MAPK MPK3/6, are also involved in this process to inhibit stomatal development and epidermal patterning (Lampard et al., 2008, 2009; Hara et al., 2009; Lee et al., 2015). In *Arabidopsis*, the polarity protein BASL serves as the scaffold for the MAPK kinase cascade, which determines asymmetric cell division (Dong et al., 2009; Zhang et al., 2015; Qu et al., 2017). In addition, proteins of BREVIS RADIX (AtBRX)-like family and Polar Localization during Asymmetric Division and Redistribution (POLAR) family have been confirmed to play key roles in stomatal development and epidermal patterning (Nunes et al., 2019).

Although bHLH, SMF, and SCRM/SCRM2 may share a close phylogenetic relationship in land plants (Liu et al., 2009; Ran et al., 2013; Cai et al., 2017), the diversification of stomatal patterning among different plant species suggests there may be lineage-specific stomatal developmental regulation during evolution (Qu et al., 2017). Based on functionally confirmed genes relevant to stomatal development in *Arabidopsis*, many studies on their orthologs in grasses have been conducted. Evidence has shown that in three bHLH paralogs, the function of FAMA is conserved across monocots and dicots, however, divergence existed in MUTE and SPCH (Liu et al., 2009). It may be due to the fact that two asymmetric divisions are needed for the formation of grass stomatal complexes. The paralogs of bHLH that regulate these divisions may have some functional diversity compared to *Arabidopsis* (Raissig et al., 2016; Hepworth et al., 2018). For instance, it was reported that two functional SPCH paralogs are partially redundant and BdICE1/BdSCRM2 control stomatal development in different temporal or spatial process in *Brachypodium distachyon* (Raissig et al., 2016). These important regulators may be likely to produce special epidermal patterning and stomatal morphology. Moreover, BdMUTE in specific SCs is related to its mobility across cells and the presence of SCs allows *B. distachyon* stomata greater responsiveness and better resilience to the environment (Raissig et al., 2017). Therefore, manipulating SC property in grasses may be an effective approach in enhancing photosynthetic performance and WUE for the breeding of climate resilient crops.

In grasses, HvEPF1, OsEPF1, and TaEPF1 of the major cereal crops have been functionally characterized. These EPFs inhibited GMC formation and arrest GMC development before SMC generation, causing substantial reduction of stomatal density in plants for better WUE without impacting grain yield in barley, rice, and wheat (Hughes et al., 2017; Caine et al., 2019; Dunn et al., 2019). It is interesting that gene duplication of EPFs/EFPL9s occurs in *H. vulgare*, *T. aestivum*, *B. distachyon*, and *Oryza sativa* and may be related to the bHLH functional diversity and parallel evolution of EPF signaling peptides for unequal stomatal complexes formation (Hepworth et al., 2018). Furthermore, some novel proteins for stomatal development have shown distinctive differences with their *Arabidopsis* orthologs. A series of studies suggests that SHORTROOT (SHRs) and SCARECROW (SCRs) are involved in the stomatal file specification and GMC formation in rice and maize (Kamiya et al., 2003; Slewinski et al., 2014; Schuler et al., 2018; Wu et al., 2019). Moreover, unlike BASL or POLAR in *Arabidopsis*, in maize, ZmSCAR/ZmWAVE regulatory complex, which contains Abl-interactor (Abi), Nck-associated protein (Nap), p53-inducible mRNA 121 (PIR121), and haematopoietic stem progenitor cell 300 (HSPC300) (Ibarra et al., 2005), is an initial marker of polarity, which polarize two other LRR receptors: PANGLOSS1 (PAN1) and PANGLOSS 2 (PAN2) (Cartwright et al., 2009; Facette and Smith, 2012; Facette et al., 2015). Both proteins promote polarization of the lateral neighboring protodermal cells, leading to their asymmetric division to form SMCs; their function may highlight the unique regulation of stomatal patterning in grasses. These studies provide promising perspectives to modify these genes for breeding programs toward drought tolerant crops.

## Stomatal Evolutionary Analysis Using OneKP

The large bulk of experimental evidences and some unsolved questions led to the potential evolutionary bioinformatics solutions for better understanding of the stomatal distinction between eudicots to monocots and for possible answers that would link high WUE and speedy grass stomata. New gene functions are generally considered to have arisen from gene duplications (Force et al., 1999). The expansions or contractions of gene family contribute to the dynamic evolution of metabolism, physiological regulation and signaling networks (Hanada et al., 2008). Since functional diversified paralogs of bHLHs (Liu et al., 2009; Raissig et al., 2016) or EPFs/EPFL9 (Hepworth et al., 2018) were found in grasses, it is necessary to figure out the relation between the stomata-associated gene duplications and different stomatal structures, especially the grass stomata. Combined comparative genetic analysis of known gene families involved in stomatal development and epidermal patterning, the distribution of these gene families may reveal the difference between basal angiosperm stomata, eudicot stomata, and the unique nature of grass stomata (Figure 1).

The release of one thousand plant transcriptomes (OneKP) provides the possibility to explore the evolution, duplication, and expansions of major gene families in large numbers of evolutionarily significant plant species (Leebens-Mack et al., 2019), in which they sequenced the vegetative transcriptomes of 1,124 species. Although it is difficult to include samples from every growing stage or from different environments, there were 80–90% universal genes conserved in the project across the *Viridiplantae*. Furthermore, gene family sizes in these transcriptomes have a significant correlation (*r* = 0.95) with those limited numbers of fully sequenced genomes (Leebens-Mack et al., 2019). In addition, cdhit (v.4.5.7) and HMMER (v.3.1b2) were used to estimate gene-family size across OneKP dataset. Sequence files for each gene family can be downloaded in OneKP Orthogroups Extractor with a valid gene identifier from one of 31 released genomes^1^, which facilitates the analysis of gene duplication across a wide range of species.

The distribution of gene families related to stomatal development may reveal the unique function of grass stomata with the analysis of gene families of 411 monocots and eudicots. Among these species, graminoid stomata has been distinguished from other eudicots or monocots on the basis of previous classification (Abbe et al., 1951; Frynsclaessens and Van Cotthem, 1973; Rudall et al., 2017; Nunes et al., 2019). Most of the 108 monocotyledonous species have paracytic or tetracytic stomata, but some species in *Alismatales, Dioscoreales, Liliales*, and *Asparagales* have anomocytic stomatal type (Supplementary Table S1). The number of each gene family member of these species has been counted and the gene duplication of species were subjected for Xgboost analysis (Figure 1B). We found that SMFs, SHRs, PANs, SCRMs, SCRs, TMMs, POLARs, and SCAR/WAVE are the important gene families (Figure 1B), which take part in the stomatal file specification and SMC formation and polarization essential for lineage specific stomatal developmental regulation.

**FIGURE 2:**
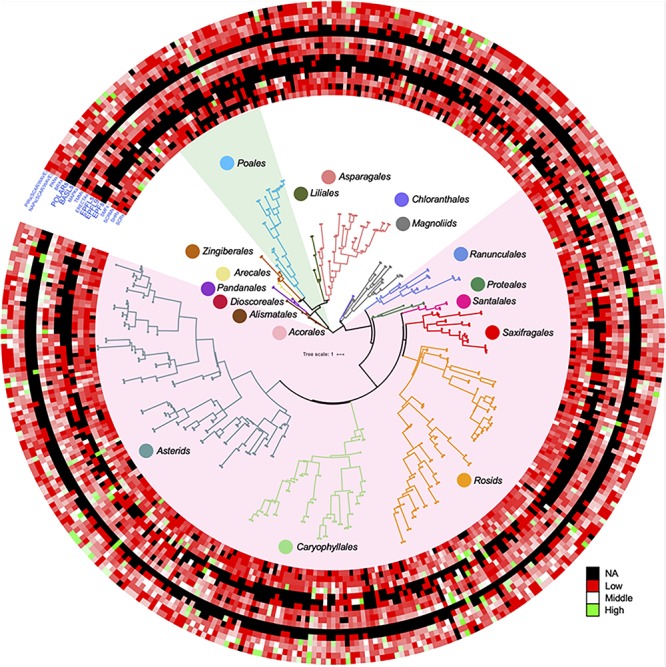
Gene duplication of key gene families for stomatal development and epidermal patterning in a large range of angiosperms. ASTRAL-II15 (v.5.0.3) was used to construct the species tree in OneKP project (https://doi.org/10.25739/8m7t-4e85), 411 eudicots and monocots were employed in this study according to (Zhao et al., 2019). The datasets of each predicted gene family distribution across these species were added on the outside of tree by iTOL (https://itol.embl.de) (Letunic and Bork, 1988). The light green and light red shades represent the *Poales* clade and eudicots, respectively. Color circles represent different clades in the phylogenetic tree. The color scale shows the number of gene family members from high (green) to low (red), and black represents a missing value. SCR, SCARECROW; SHR, SHORTROOT; SCRM, inducer of CBF expression; SMF, SPCH&MUTE&FAMA; EPF, epidermal patterning factor; EPFL, EPF-like; ERECTA, LRR receptor-like serine/threonine-protein kinase; TMM, too many mouths receptor-like protein; MAPK mitogen-activated protein kinase; BASL, breaking asymmetry in the stomatal lineage; POLAR, localization during asymmetric division and redistribution; BRX, brevis radix; PAN, PANGLOSS; NAP, Nck-associated protein; PIR, p53-inducible mRNA.

**FIGURE 3:**
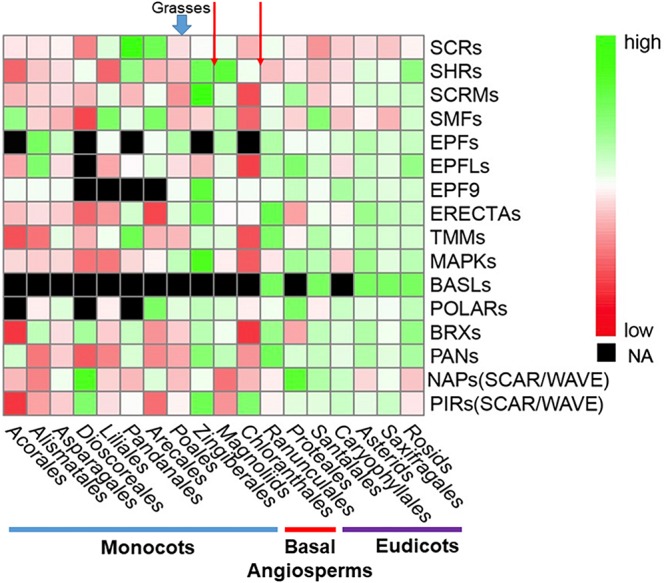
Comparison of gene and gene family distribution for stomatal development and epidermal patterning in a large range of angiosperms. Average gene-family size was counted for each clade. The heatmap was generated by pheatmap (R package) from predicted putative protein sequences of each gene family and values have been scaled in the row direction. The order of clades is arranged according to the phylogenetic tree in Figure 2. The color scale shows number of gene family members from high (green) to low (red), and black represents a missing value. Red arrows at the top of the figure represent the boundary between eudicots and basal angiosperms and monocots and basal angiosperms, respectively. Abbreviations are the same as Figure 2.

In order to show distributions of gene family directly, a phylogenetic tree was constructed for 411 OneKP species. The tree has presented all the numbers of each gene family in the representative Orders of angiosperms (Figure 2). Based on the phylogeny, there is an evolutionary transition from species of Chloranthales and Magnoliids as the basal angiosperms with kidney-shaped GCs (Pillitteri and Torii, 2012) to more complex stomata in monocots and eudicots. The most obvious gene family is BASLs, due to its absence in most eudicots and all monocots, indicating that there may be a unique polarity control of grasses (Vatén and Bergmann, 2012). Moreover, the absence not only occurs in transcriptomes, but also in sequenced and assembled plant genomes^2^. Although both BASL and POLAR drive stomatal file division, they function in two different cell fates: a meristemoid to a stoma and a SLGC to a pavement cell (Zhang et al., 2015; Houbaert et al., 2018). The sequential distinction of BASL and POLAR may explain the absence of BASL but presence of POLAR in monocots. Furthermore, the gene duplication analysis shows significant differences in major taxon (Figure 3). EPFs/EPFLs/EPFL9 are not found in some monocot clades, such as *Dioscoreale* and *Pandanales*, but are present in grasses. It appears that BASLs only exist in eudicots and there is at least one BASL homologue in most eudicot clades, except for *Caryophyllales*.

We then used OneKP to compare gene duplications of these gene families between grasses and other angiosperms. For exploring the evolutionary relationship of these gene families, Maximum Likelihood trees (Supplementary Figures S1–S10) have been constructed via PROTGAMMAWAGF model in RAxML (v8.2.12) (Stamatakis, 2014). Gene family distributions and taxon information for all species are summarized in Supplementary Table S2. In summary, we found some key gene families related to stomatal development have certain levels of different distribution in grasses in contrast to eudicots, especially regarding the function in cell division polarity and initial cell distribution. Their existence and duplication may provide grasses some functions to acquire unique stomatal morphology. However, the main limitation in this study is that the number of species only represents a tiny fraction of the total number of more than 300,000 angiosperms. Therefore, further investigation is required when there are sufficient numbers of high quality genome assemblies (i.e., 1,000) across the major orders and families of angiosperms.

## Stomatal Opening and Closure in Grass

In addition to stomatal morphological structure and development, many genetic and environmental factors are involved in stomatal response (Lawson and Vialet-Chabrand, 2018). Light (Shimazaki et al., 2007; Babla et al., 2019), CO_2_ (Engineer et al., 2014; Kolbe et al., 2018), GC membrane transport (Blatt, 2000; Hanba et al., 2004; Sade et al., 2010; Perrone et al., 2012; Lawson and Blatt, 2014), abscisic acid (ABA), reactive oxygen species (ROS), nitric oxide (NO), Ca^2+^, and pH signaling (Yang et al., 2005; Bussis et al., 2006; Hettenhausen et al., 2012; Chen et al., 2016; Wang et al., 2018), and mesophyll photosynthesis (McAusland et al., 2016; Lawson and Vialet-Chabrand, 2018) all determine the speed and magnitude of stomatal movement. Moreover, many environmental stimuli regulate stomatal opening and closure via coordinated cell signaling and membrane transport activities (Kollist et al., 2014).

Stomatal opening is tightly regulated by light (Shimazaki et al., 2007). For instance, when GCs are illuminated with blue light, photoreceptors phytochromes (PHOTs) are triggered (Kinoshita et al., 2001; Christie, 2007) to directly phosphorylate another protein kinase Blue Light Signaling1 (BLUS1), which indirectly conveys the signal to type 1 Protein Phosphatase (PP1) (Takemiya et al., 2013; Takemiya and Shimazaki, 2016). Furthermore, a Raf-like protein kinase, Blue light–dependent H^+^-ATPase Phosphorylation (BHP), bound to BLUS1 forms a signaling complex with PHOTs to phosphorylate plasma membrane H^+^-ATPase (Hayashi et al., 2017), driving H^+^ pumping and causing the hyperpolarization of the plasma membrane, activation of inward rectifying K^+^ channels, and water uptake for stomatal opening (Shimazaki et al., 2007; Marten et al., 2010; Babla et al., 2019). Blue light is one of the most influential stimuli triggering osmotic potential change and stomatal opening in grass and eudicots (Johnsson et al., 1976; Grantz and Assmann, 1991; Inoue et al., 2008). Interestingly, the PHOTs locate in different sites for light perception and phototropic bending in grass coleoptiles, but the response position is more independent in dicots (Yamamoto et al., 2014). Therefore, more research work should be conducted to investigate the different underlying mechanims between the grasses and eudicots in the key gene families (e.g., proton pumps, photoreceptors, protein kinases and phosphatases) that govern the light-induced stomatal opening.

The drought hormone ABA is one of the major signals that trigger stomatal closure (Hamel et al., 2006; Kollist et al., 2014). In *Arabidopsis*, ATP-binding cassette (ABC) transporters (AtABCG25, AtABCG40) regulate transmembrane ABA flux (Kuromori et al., 2010). Then the ABA receptors, Pyrabactin Resistance 1 (PYR)/PYR1-Like (PYL)/Regulatory Component Of Aba Receptor (RCAR) perceive ABA (Melcher et al., 2009; Cutler et al., 2010; Umezawa et al., 2010) and bind to protein phosphatases type 2Cs (PP2Cs) (Ma et al., 2009; Park et al., 2009) to activate the SNF1-Related Kinase 2s (SnRK2s) (Hauser et al., 2011). The most important member of SnRK2s is Open Stomata 1 (OST1/SnRK2.6), which directly interacts and stimulates the S-type as well as the R-type anion channels (Geiger et al., 2009; Imes et al., 2013). The activation of anion channels results in the extrusion of anions and causes the depolarization that triggers the opening of outwardly rectifying K^+^ channel for K^+^ efflux from GCs for stomatal closure (Weiner et al., 2010; Munemasa et al., 2015; Murata et al., 2015). Current evidence shows that ABA controls grass stomatal movement in a similar mechanism with some differences in the ABA concentrations and specific gene expression in GCs for eudicots and SCs for grasses and sustrates for the ion channels (Matsuda et al., 2016; Wei et al., 2014; Mega et al., 2019; Wu et al., 2019). It was found that the reciprocal responses and concentration gradient of ABA in GCs and SCs is likely to trigger fast stomatal opening and closing in grasses (Nunes et al., 2019). In *Arabidopsis*, a typical eudicot, AtSLAC1 anion channel is active in chloride-based media (Geiger et al., 2009), but its homologs AtSLAH2 and AtSLAH3 require exogenous nitrate for channel opening in oocyte and GC systems (Geiger et al., 2011; Maierhofer et al., 2014). However, OsSLAC1 was confirmed as a nitrate-selective channel in rice (Sun et al., 2016) and ZmSLAC1 and HvSLAC1 also have similar selectivity for nitrate over chloride (Qi et al., 2018; Schäfer et al., 2018). However, it is still unclear whether key genes such as SLACs in grass stomata are the key determinant for the fast stomatal closure without functional completmentation in knockout mutants of eudicots.

CO_2_ mediated stomatal closure has also been well-investigated (Kolbe et al., 2018). In the short-term, elevated concentration of CO_2_ (eCO_2_) inhibits stomatal opening and leads to stomatal closure. CO_2_ enters GCs through aquaporins (PIPs), then interacts with the carbonic anhydrase (CAs) (Gray et al., 2000; Lake et al., 2002). The interaction accelerates bicarbonate (HCO_3–_) formation to activate anion channels for stomatal closure (Negi et al., 2008; Tian et al., 2015). In the long-term, exposure to elevated CO_2_ stimulates the activity of secreted extracellular protease (CRSP), which evokes EPF2 and causes the reduction of stomatal density, thus further reducing stomatal conductance (Doheny-Adams et al., 2012; Engineer et al., 2014). However, CAs showed evidence of different effects on stomatal characteristics in *Arabidopsis* and grasses (Engineer et al., 2014; Kolbe et al., 2018). It is still unclear whether key genes such as CAs controlling stomatal CO_2_ sensing and signaling in grass stomata are different from those in eudicots and whether they are the key determinant for the fast stomatal opening and closure in grasses.

## Breeding Crops With Highly Water Use Efficient Stomata

Drought tolerant crops have the capacity to mitigate the damaging impacts of water deficit and allow plants to recover after rehydration (Morgan, 1984; Juenger, 2013). Both stomatal and non-stomatal controlling mechanisms are needed to cope with variable soil water status, which has been confirmed in a broad range of eudicot crops (Li et al., 2012; Wen et al., 2012; Tardieu et al., 2018; Gorthi et al., 2019).

The big dilemma for breeding crops with WUE is that under water stress, plants generally reduce their stomatal conductance (*g*_*s*_), which also reduces net photosynthetic rate (*A*), biomass, and yields (Tardieu et al., 2018). How can we improve WUE of crops and maintain yield under water deficiency? Recently, one study showed that without stomatal response to water stress, yield decreased by 76% in soybeans (Gorthi et al., 2019). In tomatoes, green light induced significant decreases in *g*_*s*_, and increased WUE and maintained a relatively high photosynthetic capability under short-term drought stress (Bian et al., 2019). Overexpression of aquaporin in tomatoes (Sade et al., 2010) and grapevines (Perrone et al., 2012) increased WUE under both optimal and water stress conditions. For stomatal development, it was shown that bHLHs are required for the development of soybean stomata, revealing the relationship between the accumulation sequence of GmSMFs and the initial growth stage of mature GCs (Danzer et al., 2015). Despite the success in breeding drought tolerant crops, most horticultural crops and cash crops are eudicots that do not have stomata similar to those in the crops of the grass family. It may be useful for the engineering of complex grass stomatal structure into the eudicot crop species for better WUE, drought tolerance, and eventually higher yield and quality of agricultural produces in future climatic conditions.

## Conclusion and Future Perspectives

With the increasing water deficiency and steadily growing population, breeding crops with better water and resource use efficiency is one of the top priorities in agriculture. Here, we summarized that grasses may have outstanding advantages for WUE due to their unique stomatal structure. We compared relevant gene families to find the differences between grass stomata and other monocotyledonous and dicotyledonous stomata. We found that gene duplication or absence of some gene families may contribute to the unique structure in grass stomata as most of their functions are for stomatal file specification and SMC formation and polarization (Figures 1–3). We reviewed the differential stomatal morphology, developmental mechanism, and GC signaling in monocots and eudicots. We also compared key factors and underlying mechanisms affecting stomatal opening and closure for WUE in grasses and eudicots. Therefore, manipulation of genes responsible for stomatal structure and development in crops of the grass family could be an effective approach to enhance photosynthetic performance and WUE for the breeding of climate resilient crops. It might also open opportunities for future genome editing and modification of these genes to change the stomatal complex of eudicots, accounting for the majority of food crops, to achieve a better WUE for sustainable food production. Understanding the prominent adaptability of grass stomata under drought stress is likely to provide breeding guidance for other crops.

## Author Contributions

Z-HC conceived and designed the research. YW conducted the literature search and bioinformatics and evolutionary analysis. YW and Z-HC wrote the manuscript.

## Conflict of Interest

The authors declare that the research was conducted in the absence of any commercial or financial relationships that could be construed as a potential conflict of interest.

## Footnotes

1 http://jlmwiki.plantbio.uga.edu/onekp/v2/

2 http://plants.ensembl.org/
