# Supplementary material for: Does Molecular and Structural Evolution Shape the Speedy Grass Stomata?
Source: Front Plant Sci. 2020 Apr 21;11:333. doi: 10.3389/fpls.2020.00333 (PMC7186404; doi:10.3389/fpls.2020.00333)
Supplement: Supplementary file 1 [file Presentation_1.pptx]

## Slide 1
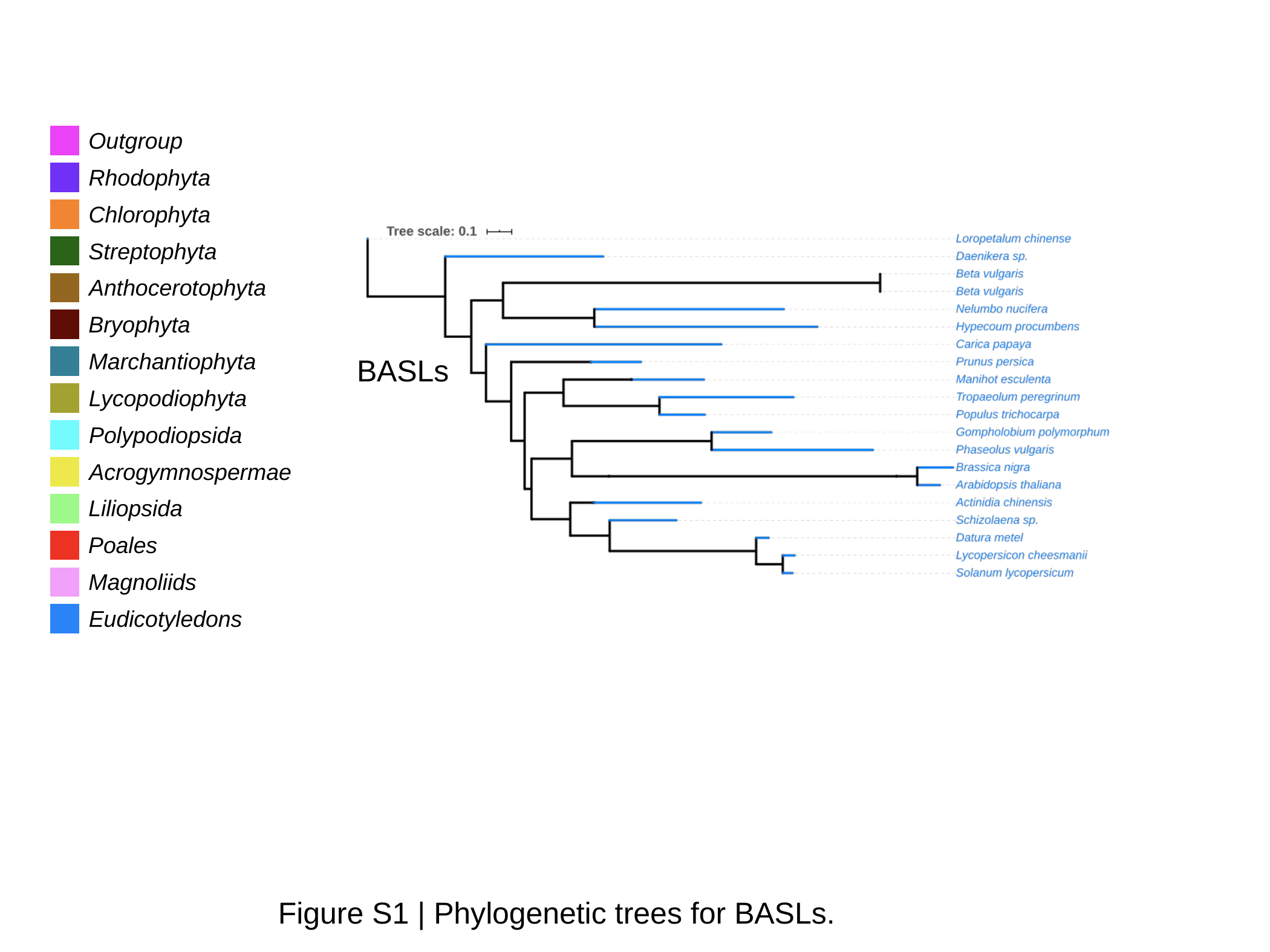

Outgroup
Rhodophyta
Chlorophyta
Streptophyta
Anthocerotophyta
Bryophyta
Marchantiophyta
Lycopodiophyta
Polypodiopsida
Liliopsida
Poales
Magnoliids
Eudicotyledons
Acrogymnospermae
BASLs
Figure S1 | Phylogenetic trees for BASLs.

## Slide 2
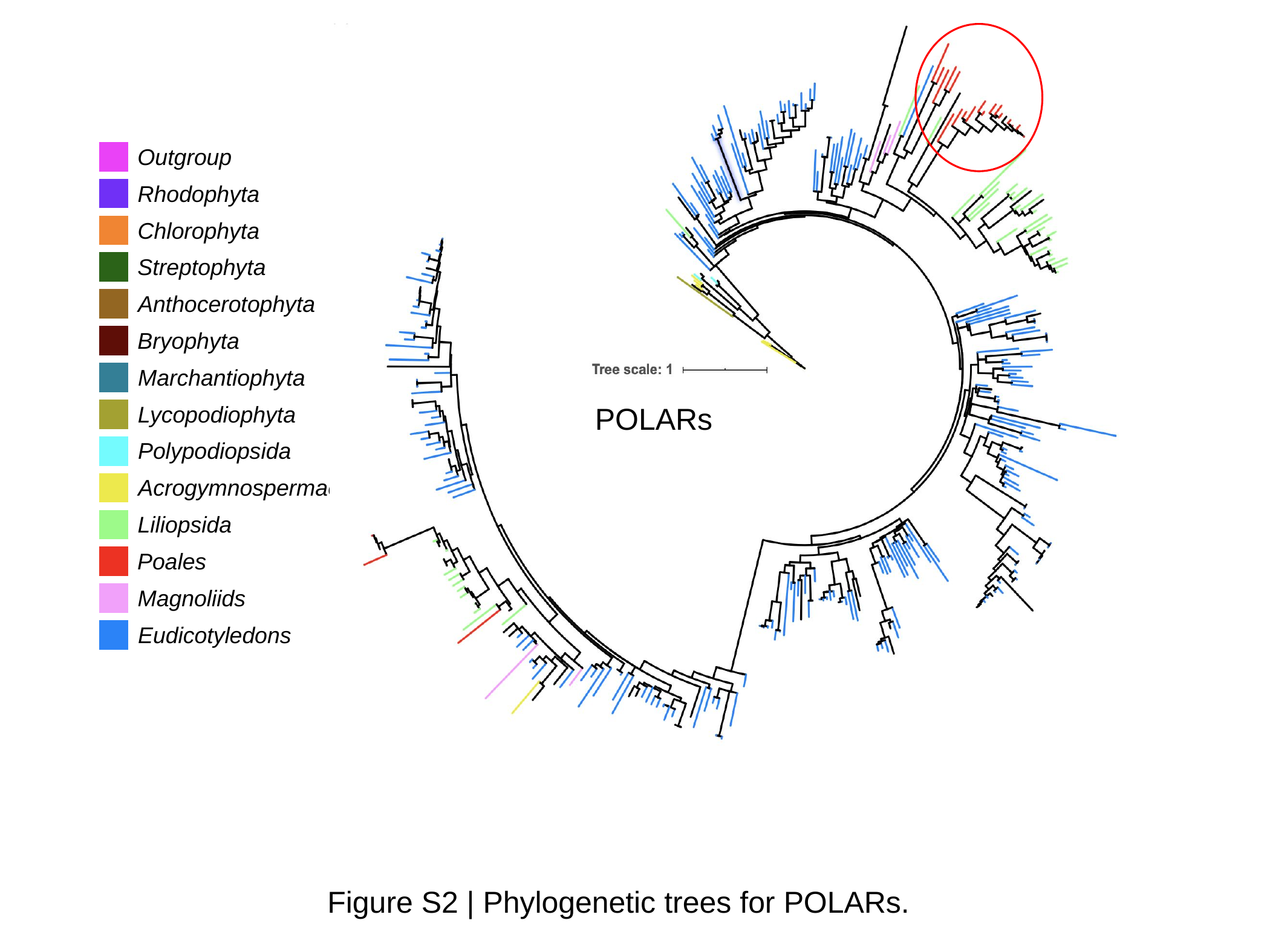

Outgroup
Rhodophyta
Chlorophyta
Streptophyta
Anthocerotophyta
Bryophyta
Marchantiophyta
Lycopodiophyta
Polypodiopsida
Liliopsida
Poales
Magnoliids
Eudicotyledons
Acrogymnospermae
POLARs
Figure S2 | Phylogenetic trees for POLARs.

## Slide 3
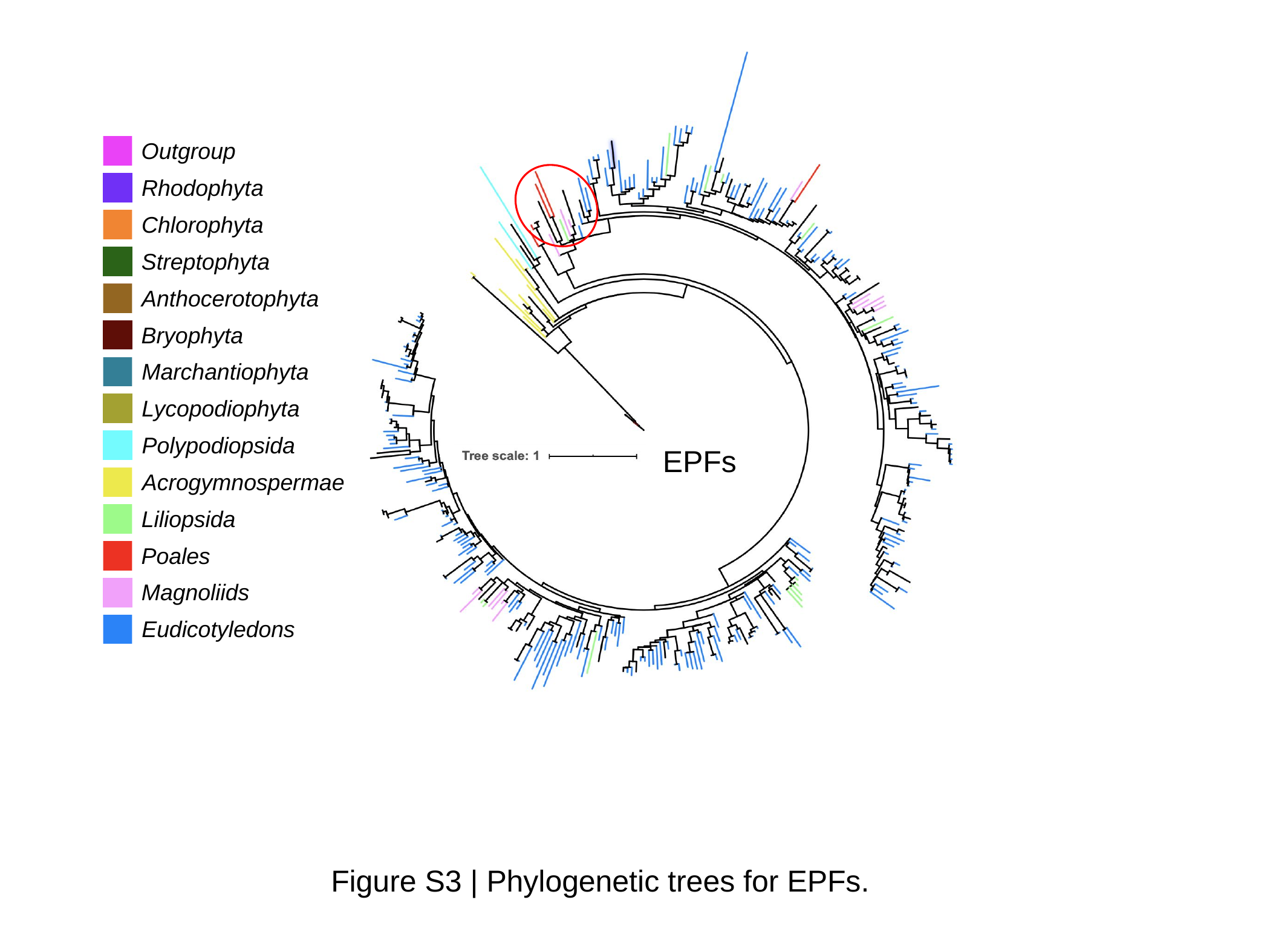

EPFs
Outgroup
Rhodophyta
Chlorophyta
Streptophyta
Anthocerotophyta
Bryophyta
Marchantiophyta
Lycopodiophyta
Polypodiopsida
Liliopsida
Poales
Magnoliids
Eudicotyledons
Acrogymnospermae
Figure S3 | Phylogenetic trees for EPFs.

## Slide 4
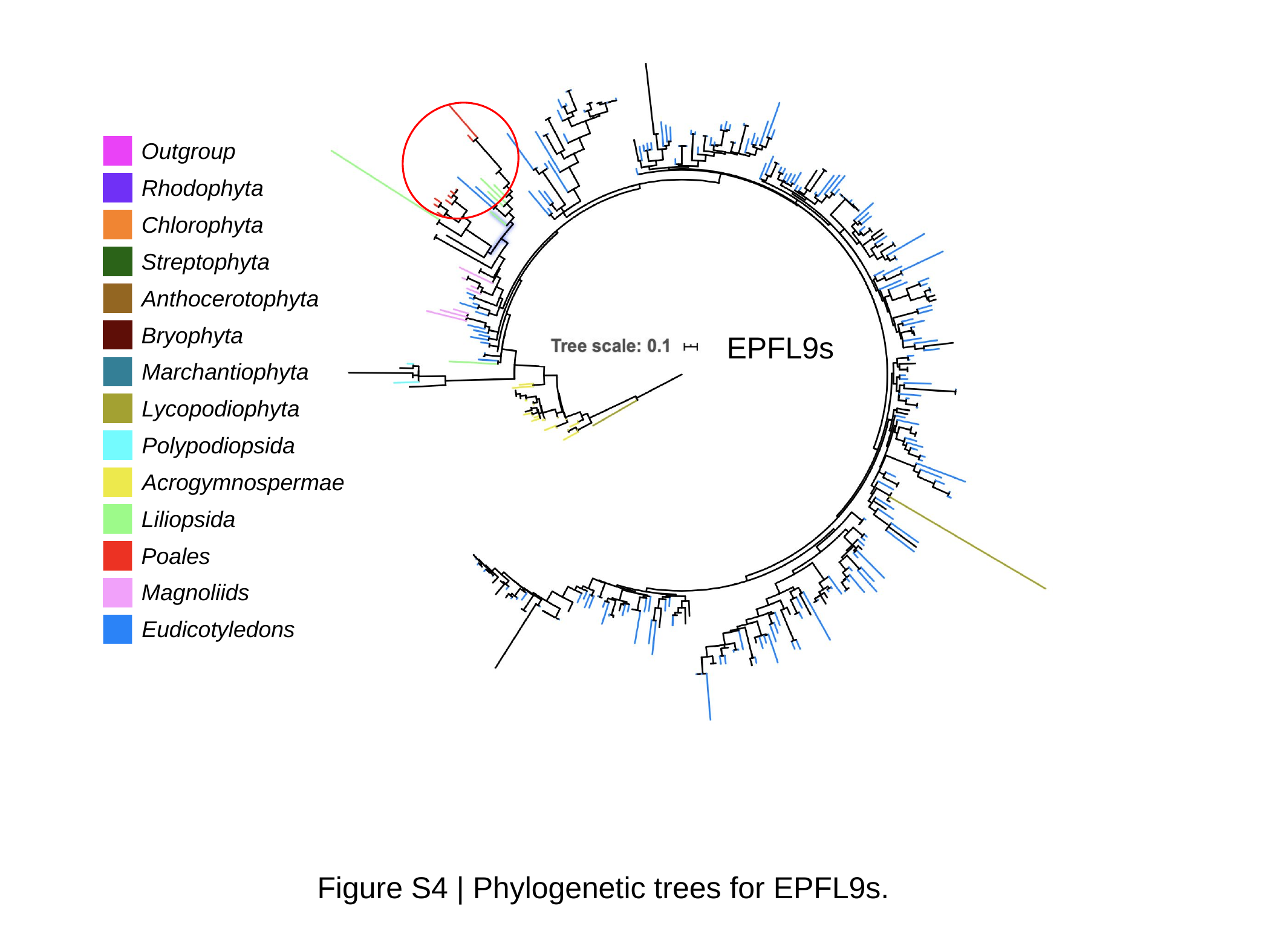

EPFL9s
Outgroup
Rhodophyta
Chlorophyta
Streptophyta
Anthocerotophyta
Bryophyta
Marchantiophyta
Lycopodiophyta
Polypodiopsida
Liliopsida
Poales
Magnoliids
Eudicotyledons
Acrogymnospermae
Figure S4 | Phylogenetic trees for EPFL9s.

## Slide 5
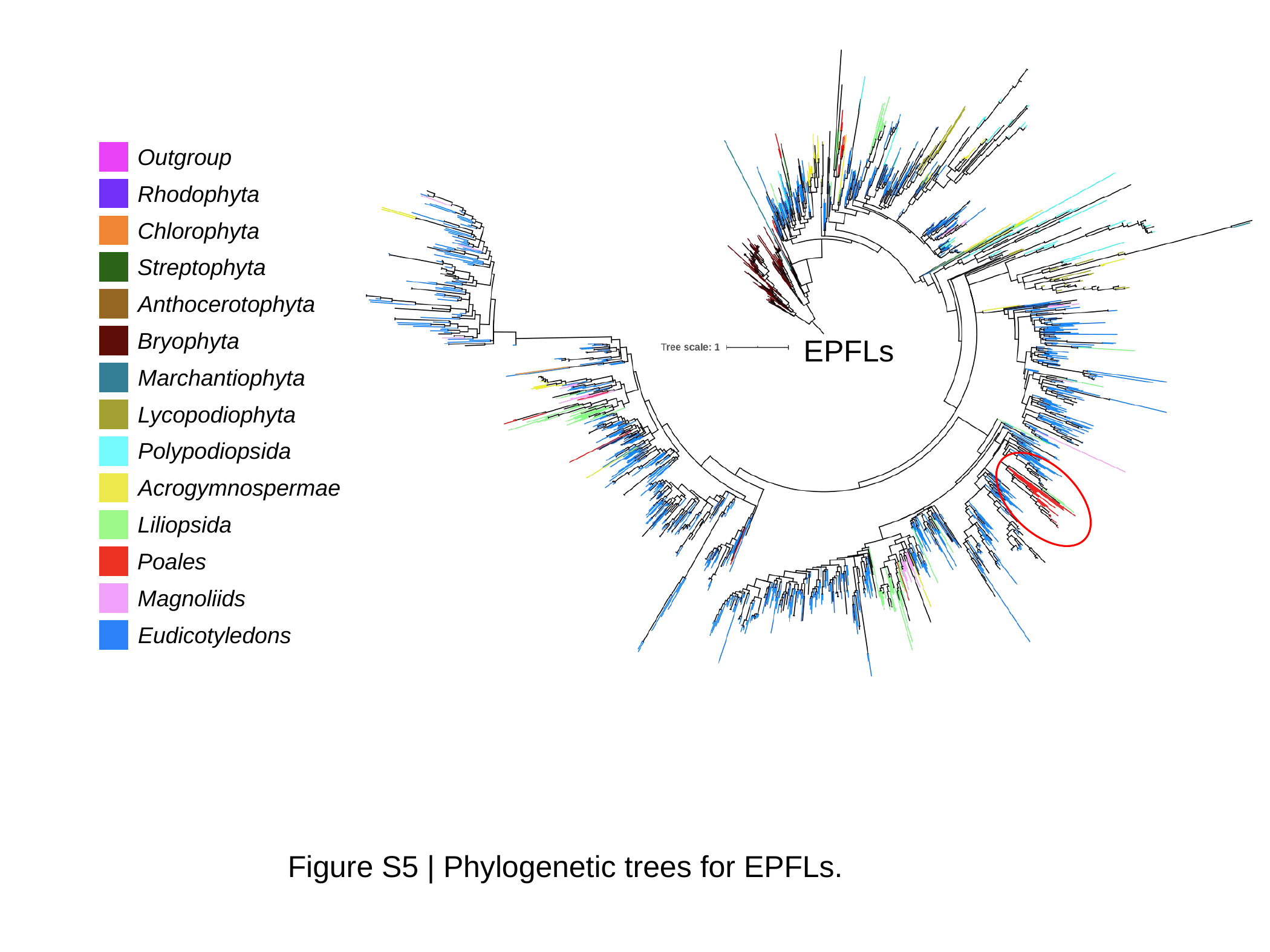

Outgroup
Rhodophyta
Chlorophyta
Streptophyta
Anthocerotophyta
Bryophyta
Marchantiophyta
Lycopodiophyta
Polypodiopsida
Liliopsida
Poales
Magnoliids
Eudicotyledons
Acrogymnospermae
EPFLs
Figure S5 | Phylogenetic trees for EPFLs.

## Slide 6
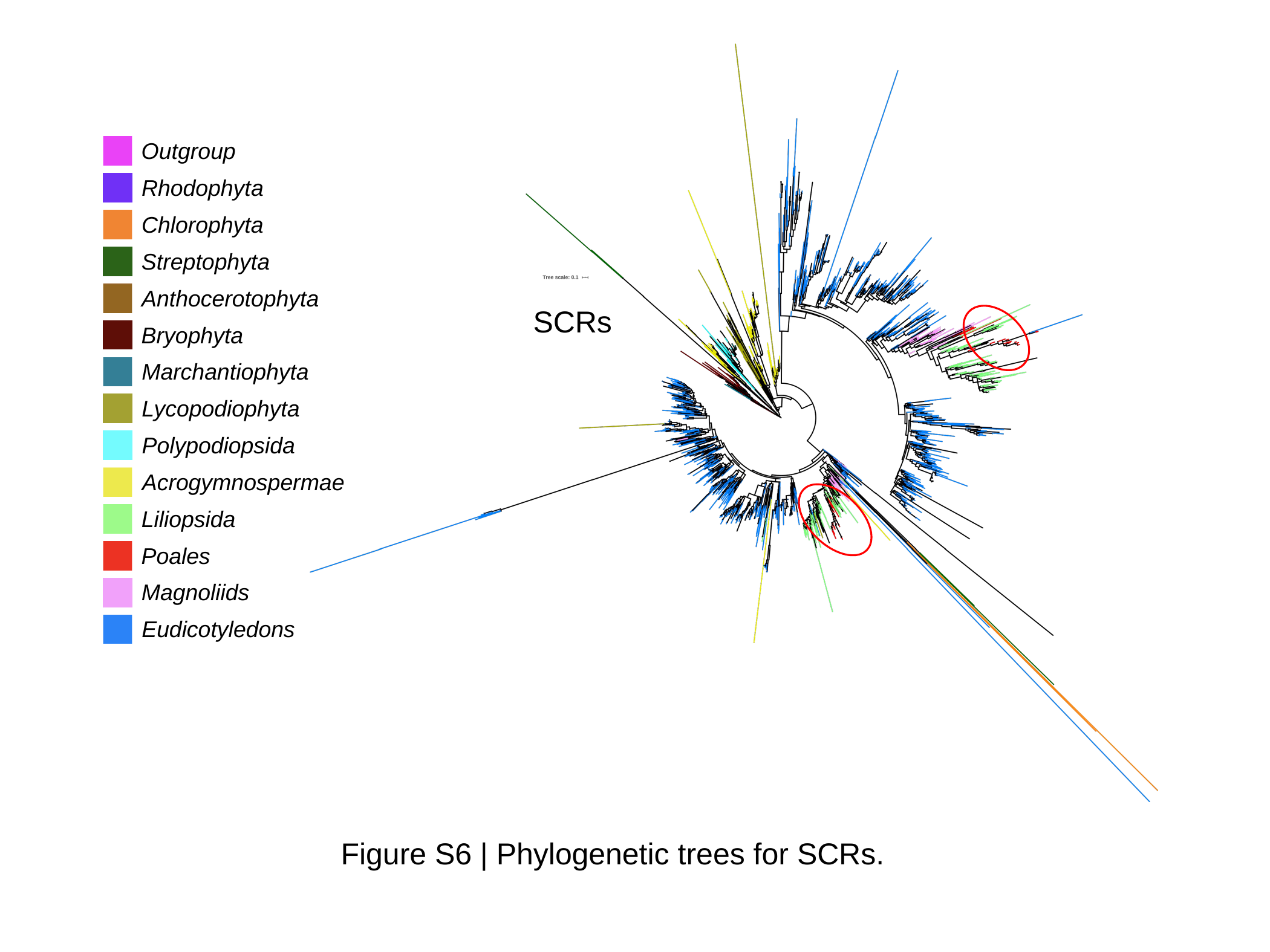

Outgroup
Rhodophyta
Chlorophyta
Streptophyta
Anthocerotophyta
Bryophyta
Marchantiophyta
Lycopodiophyta
Polypodiopsida
Liliopsida
Poales
Magnoliids
Eudicotyledons
Acrogymnospermae
SCRs
Figure S6 | Phylogenetic trees for SCRs.

## Slide 7
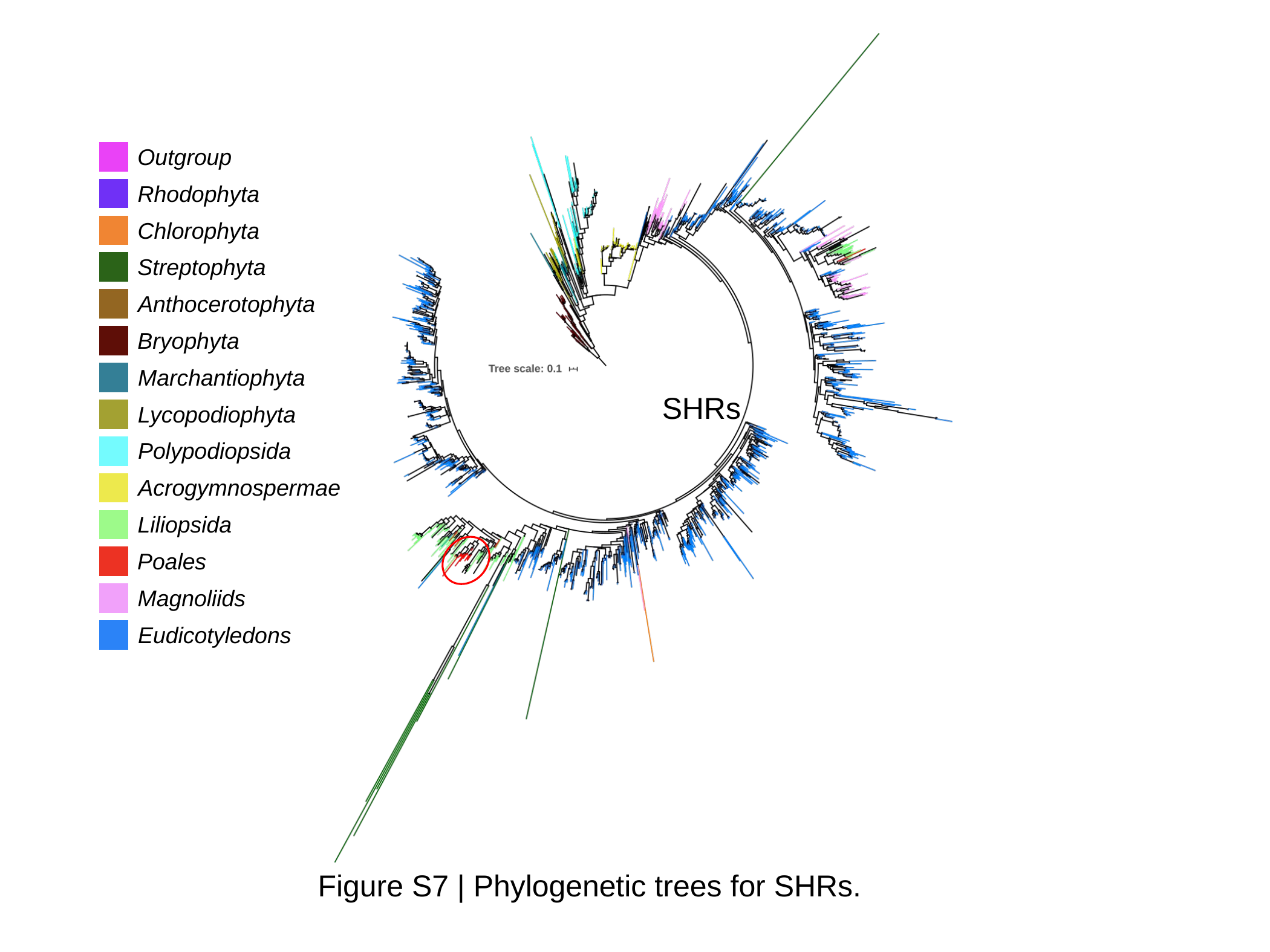

Outgroup
Rhodophyta
Chlorophyta
Streptophyta
Anthocerotophyta
Bryophyta
Marchantiophyta
Lycopodiophyta
Polypodiopsida
Liliopsida
Poales
Magnoliids
Eudicotyledons
Acrogymnospermae
SHRs
Figure S7 | Phylogenetic trees for SHRs.

## Slide 8
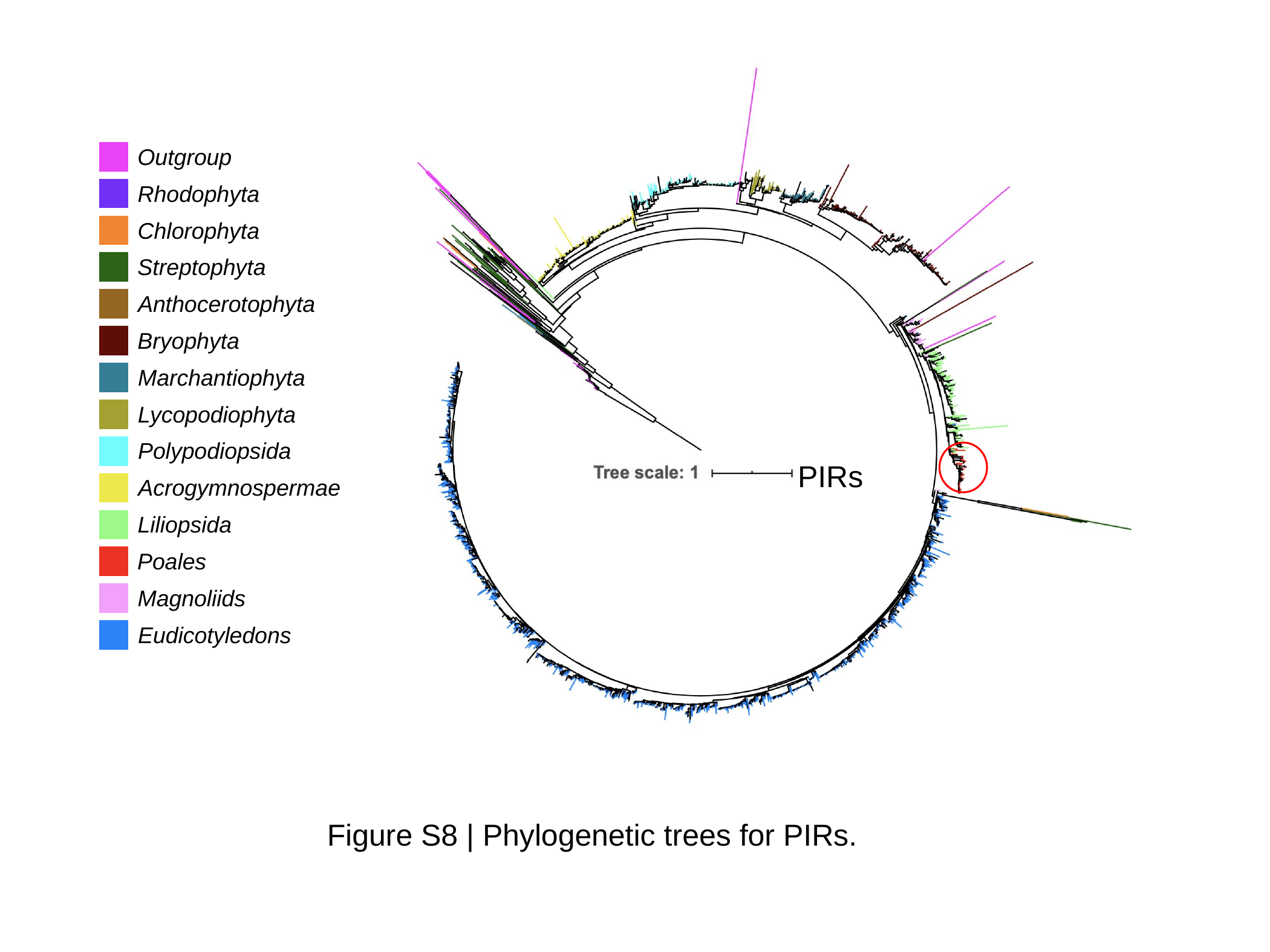

PIRs
Outgroup
Rhodophyta
Chlorophyta
Streptophyta
Anthocerotophyta
Bryophyta
Marchantiophyta
Lycopodiophyta
Polypodiopsida
Liliopsida
Poales
Magnoliids
Eudicotyledons
Acrogymnospermae
Figure S8 | Phylogenetic trees for PIRs.

## Slide 9
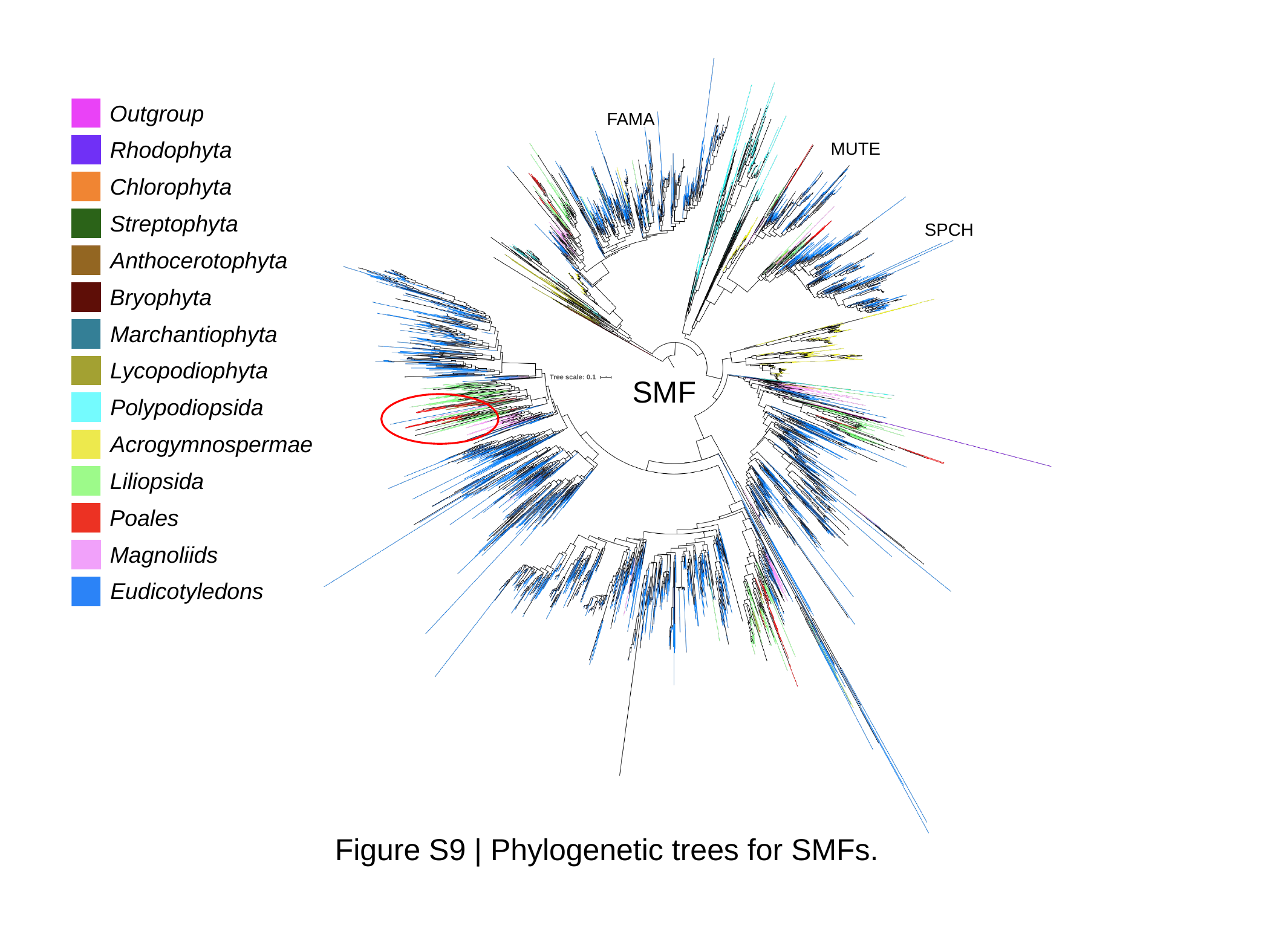

FAMA
MUTE
SPCH
SMF
Outgroup
Rhodophyta
Chlorophyta
Streptophyta
Anthocerotophyta
Bryophyta
Marchantiophyta
Lycopodiophyta
Polypodiopsida
Liliopsida
Poales
Magnoliids
Eudicotyledons
Acrogymnospermae
Figure S9 | Phylogenetic trees for SMFs.

## Slide 10
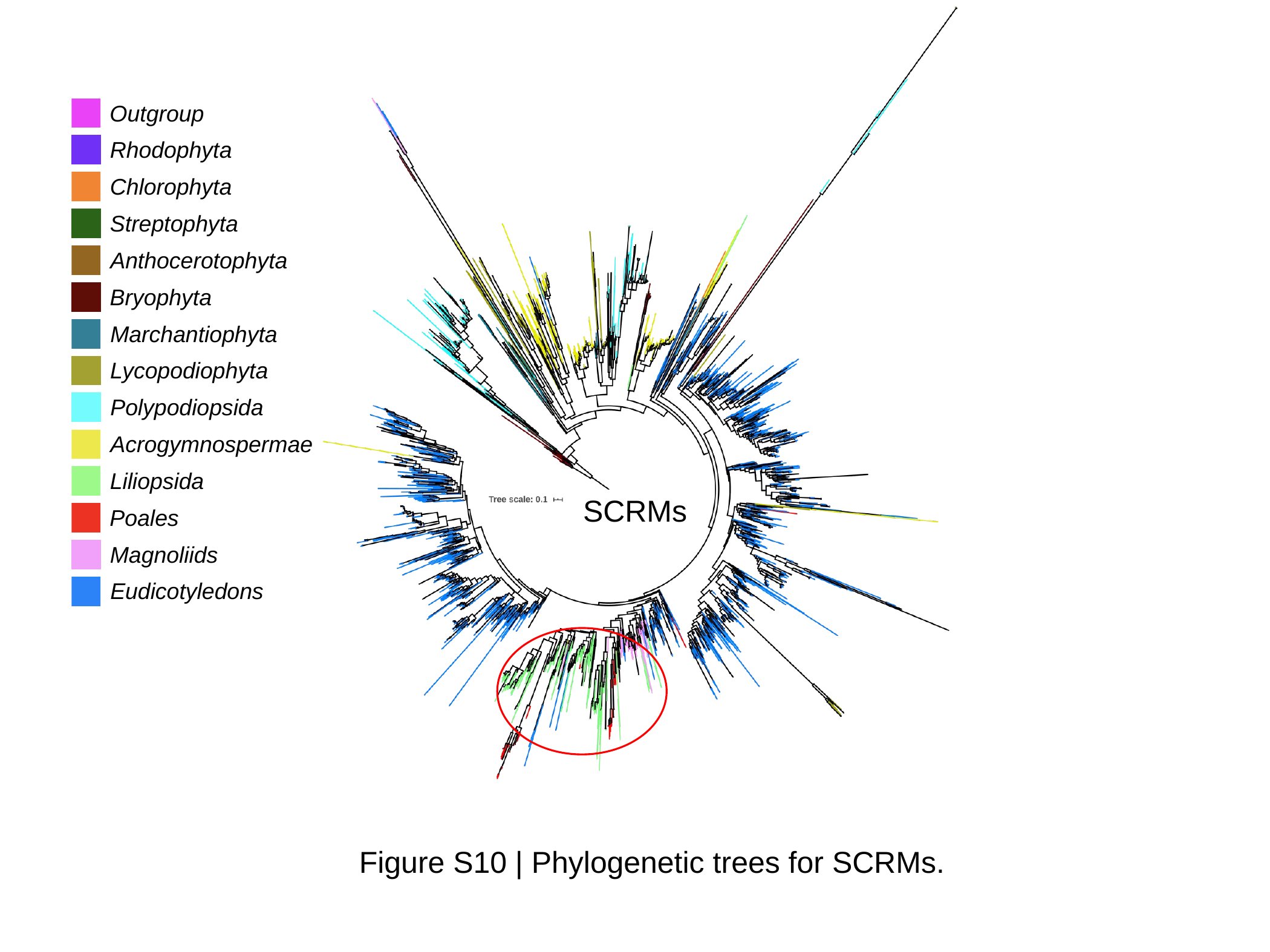

Outgroup
Rhodophyta
Chlorophyta
Streptophyta
Anthocerotophyta
Bryophyta
Marchantiophyta
Lycopodiophyta
Polypodiopsida
Liliopsida
Poales
Magnoliids
Eudicotyledons
Acrogymnospermae
SCRMs
Figure S10 | Phylogenetic trees for SCRMs.
